# Operation for Ovarian Dropsy

**Published:** 1869-03

**Authors:** Thomas P. Russell


ARTICLE XIV.

### OPERATION FOR OVARIAN DROPSY.

By THOMAS P. RUSSELL, M.D.
With Subsequent Treatment, By J. F. KELSEY, M.D.

Mrs. D., of Royalton, Wisconsin, aged 25 years, has had two children; the youngest born August, 1866. Her getting up from confinement was about six weeks, followed by the development of a tumor in the right inguinal region. I saw her first in November, 1867: laboring under an inflammation of the peritoneum, in consequence of the tumor, which had already reached the region of the stomach. Saw her again at Chicago, March 1st, 1868, in consultation with Drs. E. O. F. Roler and E. Andrews, Professor of Obstetrics and Surgery in the Chicago Medical College, who pronounced it multilocular ovarian dropsy, and told her, the only means of relief was in the removal of the tumor. She, not being prepared to be operated on, went back to her home in Wisconsin. Saw her again at her residence, December 14th, with Drs. Russell and Linde, of Oshkosh: found her much emaciated in flesh and strength; measuring 45 inches around the abdomen; had retained but little food and drink for six weeks past; vomiting from 1 to 4 times during the 24 hours; and very anxious for relief. She was told by Dr. Russell that she stood about 1 chance in 10 to live, in case of operation, and advised to take that chance; and, by her consent, would operate the next day, which was done, with the assistance, and in the presence, of Drs. Linde, Dickinson, Reiley, H. A. Frost, G. M. A. Brown, and J. F. Kelsey. An incision was made in the linea alba, about five inches in length from the navel downward; with an escape of considerable peritoneal fluid; exposing a large cyst, which was evacuated, followed by another and another, until several were evacuated, of as many colored syrupy tenaceous liquids as there were cysts. The tumor, much reduced, exposed a large, semi-solid cyst on the right side, with firm and extensive adhesions to the peritoneum, which could not be reduced. The wound was enlarged about 4 inches, directly upward: adhesions gave way, under pretty smart force, exerted for that purpose, with the fingers. The tumor turned out the pedicles; three of them, one large and two small ones, were secured with large double silk ligatures, and severed, betwixt them and the tumor, with the knife, and transfixed in the wound, at two separate places, nearly two inches apart, irrespective of their origin, the two small ones together in the lower part of the wound. The wound was then closed with silver sutures; and one steel pin passing through the edge of the wound, and small pedicle to keep it upon a level with the abdomen, it being cut a little short (they were plenty long before separating); the large one left sticking half an inch out of the wound, which was dressed with carbolic acid cerate, and bandaged with a broad flannel roller. She was then put to bed, having been under chloroform 2½ hours; the operation occupying 2 hours. The tumor and its contents weighed 40 ℔s.

*December 15th.*—Operation concluded at 1.30 o'clock, p.m.: patient in a great deal of pain; pulse, 130 per minute, feeble; respiration, 21: gave morphine, gr. ss. 2 p.m.: gave opium, gr. j., with ½ gr. morphine. 2.30 p.m.: very restless, with severe pain: gave tincture opium, gtts. 40. 3.30 p.m.: considerable pain; pulse, 120, feeble: gave opium, gr. j. 6 p.m.: inclines to sleep; thirsty; drinks ice-water freely: gave tincture opium, gtts. 40. 9 p.m.: pulse, 120; respiration, 21; use catheter; feels a little better: gave opium, gr. j. 11.30 p.m.: gave tincture opium, gtts. 40. *16th*—12.30 a.m.: more restless, with pain; pulse, 130; tongue coated with a brown fur, dry and swollen: gave opium, gr. j. 1.30 a.m.: gave opium, gr. j. 2.30 a.m.: has slept a little; pulse, 120; respiration, 20; says she feels some better; inclined to be wakeful: gave tincture opium, ½ teaspoonful. 7 a.m.: complains of smarting pain at seat of wound; pulse, 120; respiration, 18; use catheter; wants to be turned in bed often, which has been done gently, to her satisfaction: gave opium, gr. jss., and ordered ½ teaspoonful tincture opium to be taken every three hours. 4.30 p.m.: pulse, 120; respiration, 12; tongue moist; skin cool; voids urine herself; takes a little crust coffee, and feels quite comfortable. 9 p.m.: complains of nausea, with pain in the bowels; tenderness; pulse, 130 per minute, feeble; has voided urine 3 times since 5 o'clock: gave, in addition, opium, gr. ij. 11 p.m.: feels a little better: gave opium, gr. jss. 11.45: vomits a little water: gave sub-nit. bismuth, gr. 20; tincture opium continued. *17th*—11.30 a.m.: has vomited once; her flannels were changed yesterday, and again to-day, with considerable discharge of fetid, bloody serum from the wound; the tongue is coated with a dry, brown fur, with moist edge; lips parched, with considerable sordes on the teeth; pulse, 120; respiration, 12; is quite free from pain; takes a little beef-tea. 4.30 p.m.: nauseated to the stomach: gave sub-nit. bismuth, gr. 20, which relieved it. *18th*—11.30 a.m.: just dressed the wound for the first time; abdomen tender and slightly tumefied; great soreness in the right side, and very thirsty: tincture opium with beef-tea continued. *19th*—11 a.m.: the decomposition of the stump smells very bad, sickening her at the stomach. 10 p.m.: skin cool; tongue moist, with white fur; pulse, 120; tenderness of the abdomen increased, treatment the same. *20th*—3 a.m.: patient very restless, with flatulence of the bowels: gave an enema of permanganate of potass, gr. ij.; aqua, oj., which procured two good evacuations and relieved her. 12.30 p.m.: have just dressed the wound; less soreness; the large stump puffing out of the wound, the bigness of a man's fist; gangrenous. So far, she exceeds our most sanguine expectation towards a recovery. 11 p.m.: a little nausea: gave bismuth, gr. 20: feels a disagreeable sensation in the bowels: gave an enema of permang. of potass, gr. j.; aqua, oj.; retained, and patient feels better. *21st*—3 p.m.: just removed a large amount of dead tissue from the stump; treatment the same. *23d*—4 p.m.: wound some inflamed, with fulness of the bowels; otherwise cheerful: gave an enema of permanganate of potass; retained; takes tincture opium, in ½-teaspoonful doses, every 4 hours. *24th*—9 a.m.: considerable discharge of pus; bowels more flatulent; remove one suture, for the first, and syringe the wound with dilute carbolic acid. 3 p.m.: gave an enema of oleum terebinth., ʒj.; oleum ricini, ℥j.; starch, oj.; retained. 8.50 p.m.: bowels largely distended: gave another of soap-suds, ojss.; retained. 10.15 p.m.: another of soap, salt, and molasses; retained. 11 p.m.: another of permanganate of potass, gr. ij.; aqua, oj., which operated, discharging some flatus; vomited again, and took bismuth, sub-nit., gr. 15, immediately after, with considerable relief; tincture opium continued as before. *25th*—7 a.m.: bowels still flatulent: gave another enema of permang. potass; retained. 9 a.m.: another of the same, which operated with considerable relief. 6 p.m.: gave another enema of permang. potass; retained. 6.45 p.m.: gave another of the same, which operated. *26th*—8 a.m.: bowels still flatulent: ordered the following solution, ¼ teaspoonful to be taken every 6 hours:—Strychnia, gr. j.; nitric acid, gtts. 30; aqua, ℥ij.; alternated with ¼-teaspoonful of laudanum, if the patient should have any pain. *27th*:—She took an enema of the permanganate last night; operated nicely; bowels but slightly flatulent; removed nearly all the remaining dead tissue from the large stump; wound discharging freely; appetite increasing; patient cheerful. The temperature of the room was kept the first week ranging from 57 to 60 degrees (Fahrenheit), and well ventilated; since then, still lower. *January 2d, 1869*:—removed the sutures and pin to-day; wound looks well; patient improving; is still taking the strychnine solution, as before.

---

MICROSCOPICAL EXAMINATION OF A HEALTHY OVARY.—It is found, by M. Sappey, that the number of ovisacs and ovules contained in one healthy ovary amounts to more than 300,000; consequently, the individual would have about 700,000. He calculates that if all the ova found on the surface of the ovaries of a young woman, eighteen or twenty years of age, were to be fecundated, one woman only would be required to populate four such cities as Lyons, Rouen, Marseilles, and Bordeaux; two women only would be necessary to furnish inhabitants for a city of 1,600,000 persons.—*N. Y. Med. Record.*

